# Is Conspicuous Consumption Influenced by Cyber-Ostracism? A Moderated Mediation Model

**DOI:** 10.3390/bs13010006

**Published:** 2022-12-22

**Authors:** Chonghuan Xu, Guoqiang Yang, Yajuan Wang, Austin Shijun Ding

**Affiliations:** 1 Modern Business Research Center, Zhejiang Gongshang University, Hangzhou 310018, China; 2 School of Business Administration, Zhejiang Gongshang University, Hangzhou 310018, China; 3 Zheshang Research Institute, Zhejiang Gongshang University, Hangzhou 310018, China; 4 Institute of Consumer Behavior and Digital Marketing, Zhejiang Gongshang University, Hangzhou 310018, China; 5 Sobey School of Business, Saint Mary’s University, Halifax, NS B3H 3C3, Canada

**Keywords:** cyber-ostracism, social media use intensity, sense of control, implicit personality, conspicuous consumption

## Abstract

This study starts from the cyber-ostracism experience that often occurs during social media use. Based on compensatory control theory, we explore the mechanism by which cyber-ostracism affects consumers’ conspicuous consumption through the sense of control and also explore the moderating role of social media use intensity and implicit personality. This study used a sample of 407 social media users in China to verify the relationship between variables. The findings show that: there is a significant positive effect of cyber-ostracism on conspicuous consumption; sense of control plays a mediating role in the relationship between cyber-ostracism and conspicuous consumption; social media use intensity negatively moderates the relationship between cyber-ostracism and sense of control, and implicit personality moderates the relationship between sense of control and conspicuous consumption. The findings of the study help to reveal the formation mechanism of social media users’ conspicuous consumption, which has important theoretical significance and practical value for establishing correct consumption concepts in the social media context.

## 1. Introduction

Conspicuous consumption is an act of acquisition and possession by consumers in order to expand themselves and expect others to perceive them in the way they want [1]. Studies have shown that excessive conspicuous consumption can lead to depression, helplessness, anxiety, and even self-injurious behavior, which can seriously jeopardize physical and mental health, values, and property security [2]. Previous studies exploring the causes of conspicuous consumption in non-social media contexts are relatively mature and have analyzed external contextual factors and consumer psychological factors, which include reference group influence, brand association, the rarity of goods, and social exclusion [1,3], and consumer psychological factors, which include opposite-sex attraction, status concern, ego, materialism, and face consciousness [4,5]. In contrast, only a few studies have explored the causes of conspicuous consumption in social media contexts; as shown in Table 1, Bazi et al. [3] find that consumers interact with luxury brands on social media in order to improve status and face and achieve self-consistency. Siepmann et al. [6] argue that low self-esteem leads individuals to develop a need to display their status. However, most of these studies have been conducted from the perspective of consumer psychology, such as materialism, the pursuit of uniqueness, and self-esteem [3,7,8].

Cyber-ostracism is a new form of social exclusion derived from real social exclusion with the development of online technology [14]. As a common experience in the process of using social media, cyber-ostracism threatens users’ sense of belonging, self-esteem, sense of control, and the basic need for a meaningful existence and has a strong and lasting impact on individual psychosocial adaptation [15]. Currently, cyber-ostracism is widely available on Facebook [16], Twitter, Instagram [17], and virtual social media platforms [18,19]. Individuals who suffer from cyber-ostracism choose to withdraw from social interactions to protect themselves [20], and cyber-ostracism disrupts individuals’ emotional and psychological well-being, feel more negative emotions, and significantly increases the risk of depression [21]. Existing studies have explored the impact of cyber-ostracism on consumption behavior, but mostly from the perspective of relational needs for belonging and self-esteem [22]. Liu et al. [23] argue that the negative emotions associated with cyber-ostracism can weaken individuals’ feelings and perceptions of their surroundings and reduce their sense of control. Mandel et al. [24] find that when control is threatened, individuals will compensate through various behaviors to regain control. Conspicuous consumption allows individuals to gain recognition and acceptance from reference groups, enhance their self-image, and demonstrate prestige to others [21].

In addition, individuals’ perceptions of the external environment and emotional changes during social media use are influenced by their level of social media use intensity [25]. Social media use intensity refers to the level of engagement, emotional connection, and integration of users in different social media. Social media use intensity is effective in reducing social anxiety and increasing life satisfaction and self-esteem [26]. Compared to low social media intensity users, high social media intensity users experienced less negative emotions and lower uncertainty about the outside world after being rejected online [25]. In studies related to consumer behavior, the difference in the degree of influence brought by individual consumer differences cannot be ignored [27,28].

Implicit personality is the essential understanding of human characteristics, and it has been shown that consumers’ implicit personality can influence consumers’ behavioral decisions; entity theorists tend to choose products that express themselves without effort compared to gradualism [29]. Based on implicit personality theory, entity theorists believe that things are fixed, and they believe it is important to show and prove themselves to the outside world, so they tend to purchase showy goods that can directly and quickly enhance their status, whereas incremental theorists are more inclined to enhance their self-worth through their own efforts to learn. Therefore, in social media contexts, implicit personality can also, to some extent, explain the different consumer behavior responses that different individuals choose to adopt when faced with cyber-ostracism.

Therefore, based on the compensatory control theory, this paper proposes a mediating effect of sense of control in social media contexts, i.e., when individuals suffer from cyber-ostracism, their sense of control decreases, and individuals often respond to the resulting threat by compensatory consumption to enhance their sense of control, and they are more inclined to purchase conspicuous products that display their status and position to demonstrate their control and power. Meanwhile, this study introduces the variables of social media use intensity and implicit personality as moderators to explore their roles in the relationship between cyber-ostracism and conspicuous consumption.

The theoretical contributions of this study are as follows: (1) From a psychological perspective, the impact of cyber-ostracism on conspicuous consumption in social media contexts is explored. Since the research on conspicuous consumption in the field of social media is still in its infancy, this research extends social exclusion to social media contexts and further effectively extends the research findings on the influence of cyber-ostracism on conspicuous consumption in the field of consumer behavior; (2) Using compensatory control theory, the mediating role of a psychological resource, the sense of control, was found between cyber-ostracism and conspicuous consumption, which provides a new theoretical perspective to explain how cyber-ostracism affects conspicuous consumption and expands the research on the sense of control; (3) This study demonstrates the moderating role of social media use intensity in the effect of cyber-ostracism on individuals’ sense of control, further enriching and expanding the research on social media use intensity; (4) The moderating variable of implicit personality was introduced to consider the variability of different users’ responses to cyber-ostracism, enriching the study of implicit personality’s consumption behavior in response to cyber-ostracism under social media use scenarios.

The practical contributions of this study are as follows: (1) With the increase in the number of communication media and the prevalence of conspicuous consumption on social media, this study helps consumers to better understand their consumption behavior in social media situations and the mechanisms behind it. (2) For businesses operating through social media platforms, corporate marketers can analyze social media data to understand consumers’ cyber-ostracism experiences and implicit personality orientations, and use appropriate marketing guidance to transform the negative effects of online social rejection into positive effects that create self-empowerment and change, thereby build more lasting relationships with consumers.

## 2. Literature Review and Hypothetical Development

### 2.1. Literature Review

#### 2.1.1. Compensatory Control Theory

Compensatory control theory suggests that the pursuit and maintenance of a sense of control is an important motivation for human beings. The perception of the sense of control can alleviate the conflict between individuals and the restless status quo to a certain extent, and when individuals feel that they lack a sense of control, they opt to maintain the sense of control through other practical ways [30]. Individuals with control deficits may adopt different strategies to restore individual control, which include problem-solving, belief enhancement, and consumption compensation [31]. Some will further restore their perceived sense of control by directly enhancing their status superiority through the purchase of items with power symbols [32]. According to compensatory control theory, when users experience cyber-ostracism in using social media, it creates a conflict between ideals and reality. It weakens the individual’s sense of control, and individuals will choose to make conspicuous consumption to enhance their own power and status in order to compensate for the threatened sense of control needs.

#### 2.1.2. Conspicuous Consumption

Conspicuous consumption is a purchase behavior that expands oneself and is correctly perceived by others according to one’s own expectations [33]. Oh [34] considers that conspicuous consumption is about gaining recognition and acceptance by the reference group, enhancing oneself, and presenting identity status to others [35]. Belk [33] finds a relationship between materialism and conspicuous consumption, which is measured by the “envy” and “success” sub-dimensions of materialism. Existing research outlines the mechanism of the formation of conspicuous consumption in terms of two major aspects: external situational factors as well as consumer psychological factors. External situational factors include the influence of reference groups, brand associations, and the rarity of goods. Nabi et al. [36] show that interpersonal relationships can influence individuals’ propensity to consume conspicuously, so people usually spend their income spend on products that can show status or achievement to reference groups to gain recognition and prestige. Bharti et al. [4] argue that when individuals’ self-esteem is threatened, they compensate for their internal need for self-affirmation by seeking high-status goods.

#### 2.1.3. Cyber-Ostracism

Cyber-ostracism refers to the hostile experience of individuals feeling ignored or rejected in the online environment. Cyber-ostracism is widely available on Facebook, Twitter, Instagram, and virtual social media platforms [19]. Studies have shown that individuals do not receive sufficient feedback promptly after posting messages on social media applications such as Facebook [18], not tag photos posted on social media platforms [19], and the lack of timely responses to individuals’ statements in chat rooms can all induce an individual’s experience of cyber-ostracism. Individuals experiencing cyber-ostracism also experience negative experiences similar to those experiencing real social exclusion; the anonymity, asynchrony, and lack of social cues of social media situations make them more uncertain and delusional [14]. Cyber-ostracism can have a stronger and longer-lasting social impact on individuals’ psychology [15]. Sinha and Wang [37] prove that excluded individuals would have impulsive consumption behavior due to a lack of relationships and would prefer unplanned consumption behavior and short-term consumption satisfaction experience.

#### 2.1.4. Sense of Control

Sense of control is an individual’s self-perception of control over the external environment [31]. It is the individual’s perception and judgment of the extent to which his or her behavior can change something external, a subjective perception. A reduced sense of control can create a psychological burden on the individual [38] and generate negative coping strategies. Empirical studies have explored the relationship between the lack of control and consumer behavior from a sense of control compensation perspective [38]. Han and Rucker [39] find that individuals typically respond to the threats they face by consuming products and that those threatened by a sense of control choose to purchase goods that symbolically hold a sense of control to compensate for their self-image [40]. Wu [41] experimentally finds that the level of control affects consumers’ attitudes toward new products and that website interactivity moderates this process.

#### 2.1.5. Social Media Use Intensity

Some researchers have defined social media use intensity as a concept and metric used to describe the level of engagement, emotional connection, and life integration of individuals in the use of social media [42]. Research confirms that social media use intensity brings some positive aspects to individuals, such as reducing personal social anxiety and increasing satisfaction [43]. Furthermore, people with higher social media use intensity also receive positive feedback from others more frequently [44]. On the contrary, social media use intensity will also bring a series of negative consequences for individuals. Ding et al. [45] show that individuals with more prolonged social media use intensity would have longer exposure to superior information about others, so individuals would tend to experience social comparisons upward and thus be more prone to generate a series of negative emotions [25]. At the same time, the high self-esteem generated by high social media use intensity may reduce a person’s self-control, leading to more impulsive or indulgent behavior. In the consumer domain, studies have confirmed that different levels of social media use intensity impact consumers’ purchasing behavior [46].

#### 2.1.6. Implicit Personality

Implicit personality is an essential understanding of human characteristics. It is divided into two dimensions: entity theory and incremental theory [27]. Entity theorists believe that human traits are fixed and that human behavior is determined by such internal traits, is stable and continuous, and is virtually unaffected by external factors [47]; Incremental theorists believe that human characteristics are developmental and changeable and human behavior is influenced by various internal and external contextual factors and is time-sensitive and conditional. Murphy and Dweck [48] find that in terms of consumption preferences, entity theorists prefer products or brands that can enhance their self-image and demonstrate their excellent qualities, while incremental theorists prefer products or brands that can help them achieve self-improvement. Wheeler and Omair [28] suggest that incremental theorists are more sensitive to changes in product brands than entity theorists and that incremental theorists will change their identification with a brand when there is bad information about it, whereas entity theorists will always stick to their identification with the brand.

### 2.2. Hypothetical Development

#### 2.2.1. Cyber-Ostracism and Conspicuous Consumption

It has been shown that cyber-ostracism can threaten people’s sense of belonging and provoke individuals to seek out appropriate cues for reintegration purposes [22]. When individuals are friendless or ignored on Facebook, this can lead to a threatened sense of belonging, and they would produce a range of negative feelings [21], and these feelings caused by cyber-ostracism can fuel consumers’ need for social acceptance and status [34]. In the context of consumption, Lee and Shrum [49] suggest that neglected individuals spend more time looking at product logos when buying products, preferring the eye-catching signs of high-end clothing brands. Wen et al. [50] show that if consumers have experienced exclusion, they will choose unique products to differentiate them from the majority and will be more inclined to purchase conspicuous luxuries. Due to the characteristics of conspicuous products effectively satisfying consumers to display their uniqueness and obtain social recognition and status, in the social media context, when individuals suffer from cyber-ostracism, conspicuous consumption will be favored by individuals. Therefore, the following can be assumed:

**H_1_.**  *Cyber-ostracism has a significant positive effect on conspicuous consumption.*

#### 2.2.2. Cyber-Ostracism and Sense of Control

Schneider et al. [51] show that factors in the social environment could impact the level of sense of control. Individuals are susceptible to rejection from groups or others. Individuals who feel social rejection online can have distressing psychological experiences that lead to hindered satisfaction of the need for belonging, self-esteem, control, and existential meaning. Chen and Zheng [52] show that when there is a problem with an individual’s interpersonal relationships, it will have an impact on the individual’s sense of control, and cyber-ostracism, as a typical bad interpersonal relationship, will have a significant adverse effect on the individual’s sense of control. Pantano et al. [53] show that social exclusion can reduce consumers’ sense of control, causing anxiety and fear, and social disconnection, which can affect the shopping experience. In addition, stress can also reduce an individual’s sense of control, and the sense of control plays a role in how individuals perceive and react to stress. Cyber-ostracism, a common stressor in social media contexts, can also have an impact on individuals’ sense of control and can reduce individuals’ sense of control [23]. Thus, the following is postulated:

**H_2_.**  *Cyber-ostracism has a significant negative effect on the sense of control.*

#### 2.2.3. Sense of Control and Conspicuous Consumption

Related research has shown that a more extended period of a low sense of control can exacerbate anxiety symptoms and lead to a range of coping strategies [38]. Friesen et al. [54] show that when individuals’ sense of control decreases, they may turn to confirmation of structure, hierarchy, and status. Mb et al. [4] further confirm that a decrease in the level of control leads to an individual’s desire to make conspicuous purchases of high-status products. When individual control feels threatened, individuals tend to purchase conspicuous goods such as luxury goods because the status and position symbolized by luxury goods can help individuals gain social recognition and respect [34], thereby enhancing one’s sense of control and power and thus alleviating one’s threatened need for efficacy. When consumers feel less control during social media use because conspicuous products can provide users with a sense of status or power [55] and can protect the integrity of the self when individuals are threatened, making it possible for consumers to use it as a way to enhance their sense of control and make conspicuous consumption. Thus, it was hypothesized:

**H_3_.**  *Sense of control has a significant negative effect on conspicuous consumption.*

#### 2.2.4. The Mediating Role of the Sense of Control

Compensatory control theory suggests that a sense of control is an important tool for people to cope with uncertainty and disorder in the outside world. Individuals’ sense of control can be diminished by the uncertainty and disorder of their environment. Individuals often take other measures to intervene and regain their sense of control [56] to satisfy their basic need for certainty and order. Yoon and Kim [57] find that stress from financial strain can lead to a reduced sense of personal control. Hence, individuals seek diverse products and consumption scenarios as compensation for the lack of control. Su et al. [58] confirm that social exclusion further influences consumers’ propensity to purchase by affecting individual perceptions of control, with control playing a mediating role in both. Lee and Shrum [49] suggest that being ignored threatens individuals’ efficacy needs (sense of control and meaningful presence). Individuals choose conspicuous consumption to gain attention from peers and enhance their sense of control and presence. In social media contexts, the feelings of neglect and rejection associated with cyber-ostracism can threaten individuals’ need for efficacy [18] and reduce their sense of control. For self-adjustment and self-protection purposes, the excluded may resort to the symbolism of certain products to demonstrate their self-integrity to restore their sense of control over the outside world [59]. The sense of status or power that conspicuous products have [34], which can protect individual integrity when individuals are threatened, makes it possible for consumers to consume them conspicuously as an expression of an enhanced sense of control. Thus, we can formulate the hypothesis:

**H_4_.**  *Sense of control mediates between cyber-ostracism and conspicuous consumption.*

#### 2.2.5. The Moderating Role of the Social Media Use Intensity

Social media use intensity refers to the degree of life integration and the intensity of emotional connection of individuals to different social media during the use of social media. Social media users need to get enough feedback from each other in interpersonal communication promptly. Users will experience a stronger sense of social exclusion on the Internet if they lack feedback, thus damaging their happiness [21]. Studies have shown that social media use intensity can significantly and positively predict positive feedback online. The more vigorous the social media use intensity, the higher the frequency of positive feedback from others, effectively suppressing negative emotions such as loneliness and anxiety that develop during social interactions [60]. In the social media context, when confronted with cyber-ostracism, individuals with low social media use intensity felt more threatened by the perception of control after suffering cyber-ostracism, and the effect of cyber-ostracism on the reduction of control was more significant. Individuals with strong social media use intensity can effectively alleviate negative emotions due to more substantial positive feedback, and individuals feel less threatened when ignored or rejected by others. The attenuating effect of cyber-ostracism on the sense of control is negligible. On this basis, it was therefore hypothesized:

**H_5_.**  *Social media use intensity negatively moderates the relationship between cyber-ostracism and the sense of control, and the negative effect of cyber-ostracism on the sense of control is weaker when social media use intensity is higher.*

#### 2.2.6. The Moderating Role of the Implicit Personality

According to implicit personality theory, entity theorists do not believe that self-improvement can be obtained through effort, and they choose other means of self-improvement, such as seeking opportunities to show their positive attributes to themselves or others, while incremental theorists prefer to seek opportunities to learn and improve in order to become a better person. Murphy and Dweck [48] find that in terms of consumer preferences, entity theorists prefer products or brands that enhance their self-image and showcase their good qualities, while incremental theorists prefer products or brands that help them achieve self-improvement and learn new things. When consumers’ sense of control decreases, they tend to look for ways to change their threatened status, with incremental theorists preferring to change their status by improving their capabilities and entity theorists focusing on simple and effective ways to change or alleviate their low sense of control as soon as possible and show their status to others [61]. Conspicuous consumption can help consumers improve their self-esteem, build self-confidence, and self-worth affirmation, which can quickly enhance their image and thus alleviate the poorer status they face, so when consumers’ sense of control decreases, entity theorists will be more inclined to engage in conspicuous consumption to change their negative experience. Therefore, the following hypothesis was proposed.

**H_6_.**  *Implicit personality moderates the relationship between sense of control and conspicuous consumption. Specifically, when the sense of control decreases, entity theorists are more likely to prefer conspicuous consumption compared to incremental theorists.*

Implicit personality influences the psychological state of individuals after they suffer from cyber-ostracism and thus affects their subsequent consumption behavior. Specifically, the more an individual tends to be an entity theorist, the stronger the negative effect of cyber-ostracism on the sense of control, which leads to a higher probability of conspicuous consumption. In contrast, the more individuals tend to be incremental theorists, the weaker the negative effect of cyber-ostracism on the sense of control, and thus the lower the probability of engaging in conspicuous consumption. In summary, this paper proposes the following hypothesis.

**H_7_.**  *Implicit personality moderates the mediating role of sense of control between cyber-ostracism and conspicuous consumption, i.e., the more individuals tend to be entity theorists, the stronger the mediating role of sense of control between cyber-ostracism and conspicuous consumption; conversely, the weaker this mediating role is.*

**Figure 1:**
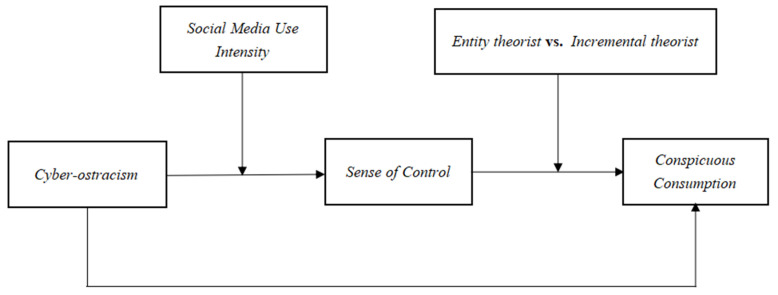
Conceptual model.

In summary, this study focuses on the effect of cyber-ostracism on conspicuous consumption and examines the mediating role of sense of control between cyber-ostracism and conspicuous consumption, as well as the moderating role of social media use intensity and implicit personality. The theoretical model is shown in Figure 1.

## 3. Methods

### 3.1. Procedures and Participants

The data of this study was collected by online questionnaire, and the questionnaire was designed on the platform of “Wenjuanxin” (the largest online questionnaire survey platform in China at present). In order to ensure the quality of the questionnaire and the validity of the data obtained, 65 users were randomly selected for pre-survey before the formal distribution of the questionnaire. The reliability and validity of the questionnaire were analyzed according to the filling of the pre-test, and the individual items in the questionnaire were revised according to the opinions of the subjects and experts. Compared with the first pre-test, the reliability and validity of the last questionnaire had been significantly improved, which accorded with the research requirements and could be issued formally.

The formal questionnaire was sent to three social media platforms with the highest user activities in China, namely WeChat, Weibo, and Xiaohongshu. We selected 600 users with conspicuous consumption experiences from these three social media platforms by convenience sampling. Next, we sent formal questionnaires to these users by e-mail (users can get a reward of ¥ 30 per questionnaire after answering). The official questionnaire was distributed from 18 May 2022 to 20 July 2022. We distributed a total of 476 questionnaires (which were not sent successfully because 124 users’ email addresses were changed or wrong) and collected and sorted out 407 valid questionnaires (69 questionnaires with larger default values were rejected), with an effective rate of 85.5% (see Table 2).

### 3.2. Measures

We adopted most of our measures from existing studies. The measures of the various constructs are shown in Table 3.

#### 3.2.1. Cyber-Ostracism

We adopted a 14-item scale from Niu et al. [14] to assess the cyber-ostracism that users encountered on social media. Each item was accompanied by a scale ranging from 1 (strongly agree) to 5 (strongly disagree). The scale has been used in Chinese university students with good reliability and validity [14]. In our study, Cronbach’s alpha was 0.974.

#### 3.2.2. Conspicuous Consumption

Based on Marcoux et al. [62] and O’Cass and McEwen [63], we adopted a 5-item scale to assess users’ conspicuous consumption behavior on social media. Each item was accompanied by a scale ranging from 1 (strongly agree) to 5 (strongly disagree). This measure translated into Chinese had good reliability and validity. The confirmatory factor analysis (CFA) indicated a good construct validity, χ^2^/*df* = 3.236. RMSEA = 0.082, CFI = 0.921, and TLI = 0.943. Furthermore, Cronbach’s alpha was 0.887 in our study.

#### 3.2.3. Sense of Control

We adopted a 3-item scale from Niemeyer et al. [64] to assess users’ sense of control in social media usage. Each item was accompanied by a scale ranging from 1 (strongly agree) to 5 (strongly disagree). This measure translated into Chinese had good reliability and validity. The confirmatory factor analysis (CFA) indicated a good construct validity, χ^2^/*df* = 2.374. RMSEA = 0.076, CFI = 0.936, and TLI = 0.952. Furthermore, Cronbach’s alpha was 0.867 in our study.

#### 3.2.4. Social Media Use Intensity

We adopted a 5-item scale from Ellison et al. [65] to assess the intensity of users’ daily use of social media. Each item was accompanied by a scale ranging from 1 (strongly agree) to 5 (strongly disagree). This measure translated into Chinese had good reliability and validity. The confirmatory factor analysis (CFA) indicated a good construct validity, χ^2^/*df* = 4.582. RMSEA = 0.074, CFI = 0.947, and TLI = 0.958. Furthermore, Cronbach’s alpha was 0.886 in our study.

#### 3.2.5. Implicit Personality

We adopted an 8-item scale from Levy et al. [66] to assess the users’ implicit personality traits. The first four items represented theorists, and the other four items represented incremental theorists. Each item was accompanied by a scale ranging from 1 (strongly agree) to 6 (strongly disagree). The four questions from the viewpoint of incremental theorists were scored reversely, and then the average score of eight questions was calculated. Referring to the practice of Plaks et al. [67], the respondents whose average score on the scale was equal to, or less than 3.0 were classified as a theorist; respondents equal to or greater than 4.0 were classified as incremental theorists; respondents between 3.0 and 4.0 were not clear enough about their implicit personality. In our study, there were no interviewees whose implicit personality view was not clear and consistent. This measure translated into Chinese had good reliability and validity. The confirmatory factor analysis (CFA) indicated a good construct validity, χ^2^/*df* = 3.742. RMSEA = 0.086, CFI = 0.952, and TLI = 0.933. Furthermore, Cronbach’s alpha was 0.889 in our study.

#### 3.2.6. Control Variables

Gender, age, education level, and monthly living expenses were selected as control variables for this study. The gender of the individuals was treated as a dummy variable. Age was measured in 4 levels. Educational level was measured in 4 levels. Monthly living expenses are measured in 4 levels.

### 3.3. Analytical Method

In this study, SPSS 23.0 and AMOS 23.0 were used to statistically analyze the results of the returned questionnaires and to determine whether the proposed research hypotheses were valid based on empirical analysis. The specific analysis methods include reliability and validity tests on the data from the pre-research, descriptive analysis, reliability analysis, correlation analysis, and regression analysis on the data obtained after the formal questionnaire research obtained after the correction of the pre-test questionnaire.

## 4. Results

### 4.1. Responses Bias Test

In questionnaire-based empirical studies, if all observed variables in a questionnaire were answered by the same subjects often triggers common method bias and affects the study results. In this study, the procedure was controlled by anonymous investigation and reversed scoring of some questions. At the same time, the Harman single-factor test was adopted to test the common method deviation. The results show that there were five factors with characteristic roots greater than 1, and the cumulative variance explained by the first factor was only 37.467%, which was less than the critical value of 50%, indicating that there was no serious common method deviation in this study.

### 4.2. Descriptive Statistics and Correlation Analysis

We conducted preliminary analyses, including descriptive statistics of the study variables by SPSS 23.0 and AMOS 23.0. The means, standard deviations, and correlation coefficients of the five variables are shown in Table 4. Cyber-ostracism was significantly negatively correlated with a sense of control and significantly positively correlated with conspicuous consumption and implicit personality. Conspicuous consumption was significantly negatively correlated with a sense of control and significantly positively correlated with the intensity of social media use. The analysis revealed a good correlation among all variables, which laid the foundation for the regression analysis that followed.

### 4.3. Confirmatory Factor Analysis

This study conducted a confirmatory factor analysis (CFA) on the measures of the key variables to test factor structure and construct validity. We modeled five factors: cyber-ostracism, conspicuous consumption, sense of control, social media use intensity, and implicit personality. The results are shown in Table 5. This theoretical five-factor model provided a reasonable fit to the data (χ^2^ = 393.446, *df* = 254, CFI = 0.975; TLI = 0.972; RMSEA = 0.037; SRMR = 0.042). Chi-square difference tests revealed that the five-factor model fits the data significantly better than several alternative measurement models. The results confirmed the theoretical five-factor model, thus supporting discriminant validity among the measures.

### 4.4. Direct Effect Analysis

We tested Hypotheses 1–3 by hierarchical regression analysis, and the results are presented in Table 6. After controlling for gender, age, education, and monthly living expenses, regression analysis revealed that: First, in Model 1, cyber-ostracism positively predicted conspicuous consumption (β = 0.406, SE = 0.050, *p* < 0.001). Therefore, hypothesis 1 was supported; Second, in Model 2, the results show that cyber-ostracism negatively predicted a sense of control (β = −0.569, SE = 0.043, *p* < 0.001). Therefore, hypothesis 2 was supported; Third, in Model 3, the results show that a sense of control negatively predicted conspicuous consumption (β = −0.304, SE = 0.056, *p* < 0.001), explaining significant additional variance in conspicuous consumption (Δ*R^2^* = 0.167, *p* < 0.001). Therefore, hypothesis 3 was supported.

### 4.5. Mediating Effect Analysis

Referring to the mediation test procedure suggested by [68] and [50], Model 4 of the SPSS macro program PROCESS was used to check the mediating role of a sense of control between cyber-ostracism and conspicuous consumption. We followed the procedure established by Baron and Kenny [69]. The research variables (excluding demographic variables) in the regression model were standardized.

As shown in Table 6, regression analysis revealed that: In Model 1, cyber-ostracism significantly and positively predicted conspicuous consumption (β = 0.406, *p* < 0.001). In Model 2, cyber-ostracism significantly and negatively predicted a sense of control (β = −0.569, *p* < 0.001). However, in Model 3, due to the intervention of a sense of control (β = −0.304, *p* < 0.001), the influence of cyber-ostracism on conspicuous consumption decreased but was still significant (β = 0.232, *p* < 0.001). Therefore, a sense of control had a partial mediating effect between cyber-ostracism and conspicuous consumption.

To confirm this result, we applied the Preacher and Hayes [70] indirect test, which applies the bootstrap method to obtain more reliable estimates. The Bootstrap method’s results showed that a sense of control could serve as a mediator between cyber-ostracism and conspicuous consumption, the indirect effect accounted for 42.373% of the total effect, SE = 0.039, and 95% CI = [0.100, 0.255]. The results showed that the sense of control plays a partial mediating role in the relationship between cyber-ostracism and conspicuous consumption. Therefore, hypothesis 4 was supported.

### 4.6. Moderated Mediation Effects Analysis

#### 4.6.1. The Moderated Mediation Effect of Social Media Use Intensity

**Figure 2:**
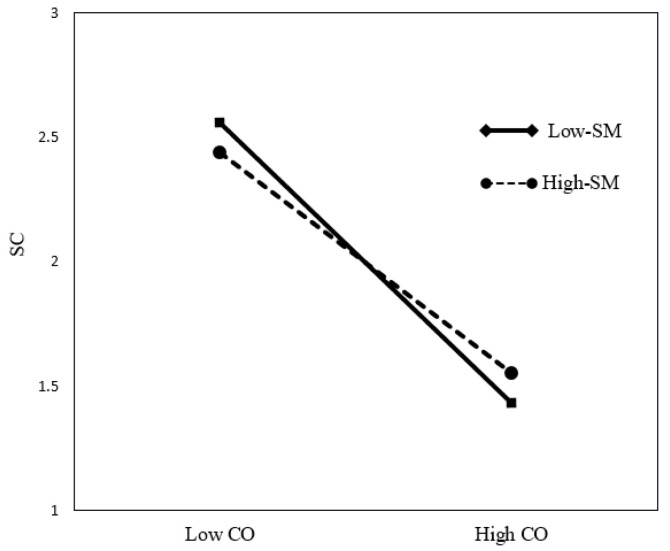
The Moderating effect of Social media use intensity (SM) on the relationship between Cyber-ostracism (CO) and Sense of control (SC).

As shown in Table 7, Regarding the moderating role of social media use intensity, the interaction term of cyber-ostracism and social media use intensity significantly predicted a sense of control (β = −0.135, *p* < 0.001; ∆*R^2^* = 0.167, *p* < 0.001) in Model 3. In Figure 2, to facilitate the interpretation of the moderating effect, a graph was drawn that distinguishes between high and low social media use intensity groups based on the average value of social media use intensity. As shown in Figure 2, there was a difference in the relationship depending on the degree of social media use intensity. Therefore, social media use intensity negatively moderated the relationship between cyber-ostracism and the sense of control, and the negative effect of cyber-ostracism on the sense of control was weaker when social media use intensity was higher. Therefore, Hypothesis 5 was supported.

To test integrative moderated mediation, we examined whether the indirect effect of cyber-ostracism on conspicuous consumption was moderated by social media use intensity. To test the conditional indirect effect, we utilized Hayes’ PROCESS program [71]. The indirect effect of cyber-ostracism on conspicuous consumption was estimated at high (+1 SD), and low levels (−1 SD) of social media use intensity with the bootstrap method. The analysis results are presented in Table 8. The results indicated that the indirect effect was significant for high social media use intensity (conditional indirect effect = −0.4503, SE = 0.0572, 95% CI = [−0.1068, −0.0243]) and was also significant for low social media use intensity (conditional indirect effect = −0.6782, SE = 0.0551, 95% CI = [−0.3033, −0.1384]).

#### 4.6.2. The Moderated Mediation Effect of Implicit Personality

**Figure 3:**
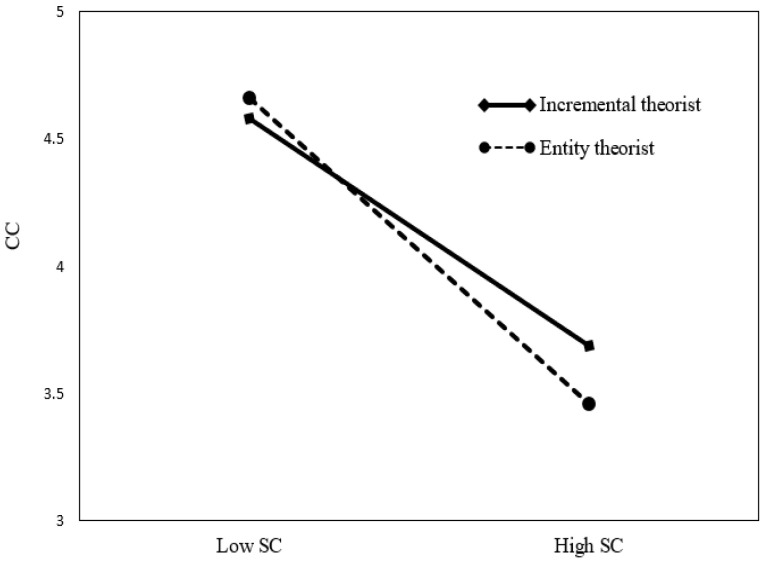
The Moderating effect of implicit personality (IP) on the relationship between Sense of control (SC) and Conspicuous consumption (CC).

As shown in Table 7, Regarding the moderating role of implicit personality, the interaction term of sense of control and implicit personality significantly predicted conspicuous consumption (β = −0.154, *p* < 0.001; ∆*R^2^* = 0.072, *p* < 0.001) in Model 6. In Figure 3, to facilitate the interpretation of the moderating effect, a graph was drawn that distinguishes between entity theorist and incremental theorist groups based on the average value of implicit personality. As shown in Figure 3, there was a difference in the relationship depending on entity theorist and incremental theorist groups. Therefore, implicit personality moderated the relationship between sense of control and conspicuous consumption. Specifically, when the sense of control decreased, entity theorists were more likely to prefer conspicuous consumption compared to incremental theorists. Therefore, Hypothesis 6 was supported.

To test integrative moderated mediation, we examined whether the indirect effect of cyber-ostracism on conspicuous consumption was moderated by implicit personality. To test the conditional indirect effect, we utilized Hayes’ PROCESS program. The indirect effect of cyber-ostracism on conspicuous consumption was estimated at incremental theorist (+1 SD) and entity theorist (−1 SD) of implicit personality with the bootstrap method. The analysis results are presented in Table 9. The results indicated that the indirect effect was significant for incremental theorist (conditional indirect effect = −0.1712, SE = 0.0741, 95% CI = [−0.1856, −0.0218]), and was also significant for entity theorist (conditional indirect effect = −0.3427, SE = 0.0674, 95% CI = [−0.2217, −0.0391]). Therefore, implicit personality moderated the mediating role of sense of control between cyber-ostracism and conspicuous consumption. Hypothesis 7 was supported.

## 5. Discussion

### 5.1. General Discussion

This study empirically investigates how cyber-ostracism affects consumers’ conspicuous consumption in the context of social media use. We tested the relationship between cyber-ostracism, sense of control, conspicuous consumption, social media use intensity, and implicit personality, supported by the sense of control compensation theory, and the results of the hypothesis testing are shown below.

H_1_ examined the main effect of cyber-ostracism on conspicuous consumption, and this hypothesis was verified through data analysis. The results of this study suggest that with the popularity of social media, which has become part of the daily life of many people, social exclusion extends to the online environment. In social media contexts, there is a significant positive relationship between the cyber-ostracism experienced by an individual and the individual’s tendency to consume conspicuously. H_2_ examined the relationship between cyber-ostracism and sense of control, and this hypothesis was supported, indicating that cyber-ostracism leads to a decrease in an individual’s sense of control. H_3_ examines the relationship between a sense of control and conspicuous consumption, and this hypothesis is also verified, suggesting that when consumers’ sense of control decreases, they are more inclined to conspicuous consumption for the purpose of improving their state of being under threat. Based on compensatory control theory, we propose H_4_ to examine the mediating role of the sense of control between cyber-ostracism and conspicuous consumption, and this hypothesis is supported, which indicates that consumers will experience different degrees of cyber-ostracism in the process of using social media, and cyber-ostracism will reduce consumers’ sense of control, and in order to compensate for their image needs, consumers are more likely to generate conspicuous consumption. In conjunction with the social media context, we chose the variable of social media use intensity as a moderating variable. H_5_ examined the moderating effect of social media use intensity on the relationship between cyber-ostracism and sense of control, and this hypothesis was tested, suggesting that social media use intensity was effective in mitigating the negative emotions felt by individuals and weakening the negative effect of cyber-ostracism on perceptions of control. H_6_ further examined the effect of implicit personality as an individual personality trait on consumption behavior, and this hypothesis was supported, suggesting that individuals with different implicit personalities would adopt different consumption behaviors to restore their sense of control when they are in a state of reduced control, and that entity theorists preferred conspicuous consumption to compensate for their sense of control compared to incremental theorist. H_7_ further examined the moderating effect of implicit personality on the mediating role of sense of control between cyber-ostracism and conspicuous consumption, and this hypothesis was also verified, implying that implicit personality moderates the mediating role of sense of control between cyber-ostracism and conspicuous consumption, i.e., the more individuals tend to be entity theorists, the stronger the mediating role of sense of control between cyber-ostracism and conspicuous consumption.

In addition, from Model 2 in Table 7, the moderating effect of implicit personality on the negative relationship between cyber-ostracism and a sense of control is not significant. The reason may be that when an individual is in a situation of self-threat, both the theorists and the incremental theorists can feel out of control and want to quickly restore their sense of control over the outside world. In practice, only the concrete performance of regaining the sense of control may be different between them. Theorists are more inclined to buy goods that can quickly improve their status and image, while incremental theorists think that the compensation of status consumption for their image is only temporary and difficult to maintain for a long time. They are more inclined to improve their ability through their own hard work to regain their sense of control [40,61].

Moreover, from Model 5 in Table 7, the moderating effect of implicit personality on the negative relationship between cyber-ostracism and conspicuous consumption is not significant. The reason may be that when being rejected by the network society, both the theorists and the incremental theorists will fight back, and they will make conspicuous consumption to deal with the rejection of the network society. However, there are still differences in the logic of behavior strategies between the two. Theorists will think that these behaviors are determined by the immutable personality traits of others, so they tend to take punitive and retaliatory measures. However, when faced with personal setbacks or failures, incremental theorists tend to adopt mastery-oriented responses, which are manifested by persisting in efforts, recognizing the specific causes of setbacks, and seeking new strategies to solve problems [48,66]. That is to say, when being rejected by the network society, both the theorists and the incremental theorists will take actions to respond and prove to improve their status, but in essence, they are conspicuous behaviors, that is, the punishment measures of the theorists and the efforts of the incremental theorists are conspicuous consumption. For example, the theorists prefer to show expensive luxury goods they directly buy through social media, while the incremental theorists prefer to show the products that they personally bought and actively participated in the efforts to make on social media.

Overall, this study provides an in-depth study of the causes of conspicuous consumption in the social media environment, which is of great value for the future development of young global consumers’ consumption perceptions and psychological qualities in the social media environment. It also has positive implications for social media marketing strategies and the construction of sustainable consumption environments for businesses around the world.

### 5.2. Theoretical Implications

First, our study makes some contributions to the literature related to social exclusion and conspicuous consumption. While existing studies have done more in-depth and comprehensive research on non-social media contexts and have explored the effects of social exclusion on conspicuous consumption, the exploration of conspicuous consumption in social media use contexts has mostly focused on individual psychological factors such as materialism, the pursuit of uniqueness, and sense of face [3,7,8,11] while ignoring the important external contextual factor of cyber-ostracism present in social media. This study builds on the existing literature by identifying the variable of cyber-ostracism in the context of the times, extending the findings of social exclusion to the online environment, and extending the study of cyber-ostracism on consumer behavior to the specific consumption behavior of conspicuous consumption. This study concludes that individuals experience different degrees of cyber-ostracism in the process of using social media. Cyber-ostracism leads to threatened individual needs, and in order to cope with the threatened state, individuals increase their attention to social status and class and then choose to consume conspicuously to satisfy their threatened psychological needs, to arouse others’ attention to themselves, and to compensate for their own image. This result enriches the research findings on the relationship between cyber-ostracism and specific consumption behaviors and their mechanisms of action in the field of consumption behavior.

Second, we have also contributed to the literature on the sense of control. In recent years, experts in the field of consumer behavior have been interested in the sense of control. Previous research on the sense of control has shown that individuals’ lack of control leads to compensatory consumption; for example, when individuals are in a state of low control, consumers are more likely to purchase high-priced luxury goods or big-ticket items [72]. The findings of this study confirm this view and extend this result to the specific consumption behavior of conspicuous consumption, expanding the research results in the field of sense of control in consumer behavior. In addition, this study proposes a new theoretical component in the field of sense of control based on previous research, that is, cyber-ostracism reduces consumers’ sense of control, and in order to compensate for their psychological needs, individuals choose to consume show-off to enhance their sense of control. This study confirms that a sense of control serves as a mediator between cyber-ostracism and conspicuous consumption, which provides a new theoretical perspective to explain how cyber-ostracism affects conspicuous consumption and helps expand the research related to the sense of control.

Third, we enriched and expanded the literature on social media use intensity. Previous studies have generally concluded that social media use intensity has negative effects on individuals and that excessive social media use intensity can increase individuals’ stress and negative emotions and even lead to depression [25,73]. In contrast, this study takes the perspective of the positive effects of social media use intensity and argues that social media use intensity can effectively alleviate the social anxiety individuals feel in the online environment, leading to differences in the degree of negative emotions individuals feel, which in turn affects the impact of cyber-ostracism on the sense of control. This study explored this boundary condition and confirmed that social media use intensity plays a moderating role in the association between cyber-ostracism and individuals’ sense of control. The impact of cyber-ostracism on the sense of control varies depending on the intensity level of social media use, which to some extent, explains the different reactions of different individuals to cyber-ostracism. When individuals with low social media use intensity experienced cyber-ostracism, the effect of cyber-ostracism on the sense of control was more significant due to the higher perceived social anxiety. However, this relationship was not significant for individuals with high social media use intensity because the social media use intensity effectively alleviated the anxiety caused by cyber-ostracism.

Finally, we enriched the research on the consumer behavior of implicit personality in response to cyber-ostracism in social media use scenarios. Previous research has demonstrated that implicit personality can influence consumers’ attitudes, choices, and decisions [74]. This study incorporates social media use scenarios, and the results show that when individuals suffer from cyber-ostracism, entity theorists develop a stronger tendency to consume in an ostentatious manner than incremental theorists. Entity theorists tend to change their negative status through conspicuous consumption without effort, whereas incremental theorists still believe that ability improvement is more important. This study confirms that different implicit personalities of consumers differ significantly in their information processing styles and concerns, providing theoretical support for further understanding individual differentiation of conspicuous consumption in social media contexts from the perspective of personality traits.

### 5.3. Practical Implications

First, the study examines the impact of cyber-ostracism on conspicuous consumption in the context of social media use. As the frequency of social media use increases, consumers inevitably suffer from online social rejection in the process of using social media. This study helps consumers to understand the causes of ostentatious consumption in the social media context and to avoid the temptation and trap of consumption by actively facing social rejection and increasing the intensity of social media use to alleviate the threat they feel so that they can make consumption decisions more rationally and return to rational consumption.

Second, we provide guidance for coping with the phenomenon of conspicuous consumption in the context of social media use. From the results of this study, it is clear that cyber-ostracism in the context of social media use can have a significant positive impact on consumers’ conspicuous consumption. Individuals are subjected to online personal chat exclusion and online group chat exclusion in the process of social media use, and these cyber-ostracisms all reduce consumers’ sense of control, which leads to consumers’ conspicuous consumption. Therefore, in the marketing process, merchants can use consumer status information to understand whether consumers are in the social situation of cyber-ostracism, and the rejected consumers should give more attention and positive feedback to alleviate the negative emotions of consumers, reduce the probability of consumers choices such as showy consumption and other transient irrational consumption behavior, and promote consumers to establish a scientific and reasonable consumption concept, so as to better establish a sustainable and long-term brand relationship with consumers.

Finally, companies should pay attention to the collection of data on consumers’ own traits. In today’s era, when social networks are prevalent, consumers often experience cyber-ostracism in the process of using social media, and in the consumer context, the sense of threat brought by cyber-ostracism will prompt them to choose the corresponding products, and for entity theorists, the quick selection and use of some showy items will be able to satisfy their psychological sense of being noticed. In contrast, incremental theorists do not feel the same way. On the contrary, incremental theorists are less concerned about conspicuous consumption, as they are more concerned about the change and improvement of their own ability; it may be easier to attract the attention of incremental theorists if they can quickly and effectively realize the benefits of conspicuous consumption on the improvement of their ability. Therefore, companies can use advertising videos and the verbal behavior of promoters to activate consumers’ incremental tendencies and turn the negative effects of cyber-ostracism into positive effects that create self-improvement and change while at the same time, collecting data on consumers’ own traits can more effectively provide different products or services to different consumer segments.

### 5.4. Limitations and Future Research Directions

First, there are some geographical limitations to the sample of this study. This study only investigated social media users in China. Given the global implications and applicability of this study, we should test whether the current effects can generally be applied to consumption in other developed countries to see if culture influences the results of the study or if it were to determine whether developments in a country have changed consumer perceptions of these issues.

Second, although this study explored the potential mechanism of the interaction between cyber-ostracism and social media use intensity on conspicuous consumption in a social media context, this study used a cross-sectional research method, although it builds on a certain theoretical foundation, can reveal the causal relationship to a certain extent, but its revealing effect is much less than that of longitudinal studies. Future research should examine whether the conspicuous consumption behavior of people with low social media use intensity increases when cyber-ostracism changes. Furthermore, the current study selected a relatively young, nationally representative sample; therefore, this finding may not be comprehensive enough. The current findings were conducted in the general population as much as possible, and future research is expected to select a larger sample for a more comprehensive scientific study.

Finally, this study only examined the relationship between cyber-ostracism and conspicuous consumption while ignoring the social exclusion individuals suffer in real life. Future research should consider both cyber-ostracism and social exclusion and explore their interaction with conspicuous consumption.

## Figures and Tables

**Table 1:** Research related to conspicuous consumption in social media contexts.

Author	Antecedent Variable	Research Paths
Pellegrino et al. [8]	materialism	materialism → social media intensity → conspicuous consumption
Islam et al. [7]	materialism	materialism → compulsive internet use → conspicuous consumption
Siepmann et al. [6]	self-esteem	self-esteem → status perceptions → conspicuous consumption
Burnasheva and Suh [9]	social media usage	social media usage → self-image congruity → conspicuous consumption
Bazi et al. [3]	status signaling	status signaling → conspicuous consumption
Bahri-Ammari et al. [10]	social comparison	social comparison → conspicuous consumption
Wallace et al. [11]	need for uniqueness	need for uniqueness → conspicuous virtue signalling → conspicuous consumption
Taylor and Strutton [12]	envy	envy → self-promotion → conspicuous consumption
Thoumrungroje et al. [13]	social media intensity	social media intensity → reliance on EWOM → conspicuous consumption
Current research	cyber-ostracism	cyber- ostracism → sense of control → conspicuous consumption

**Table 2:** Demographic characteristics (*N* = 407).

Variable	Category	Frequency	Percentage (%)
Gender	Male	196	48.16
	Female	211	51.84
Age	Less than 18 years old	21	5.16
	18~30 years old	219	53.81
	31~40 years old	135	33.17
	40 years or up	32	7.86
Education level	Technical secondary school graduation	66	16.22
	Vocation high school graduation	110	27.03
	Undergraduate	201	49.39
	Master or above	30	7.37
Monthly living expenses (RMB)	Less than 1000	39	9.58
	1000~3000	214	52.58
	3000~5000	115	28.26
	Above 5000	39	9.58

**Table 3:** Measurement items.

Construct		Measure
Cyber- Ostracism	I asked the other party to chat with me online and got no reply	Niu, et al. [14]
	When I ask for help online, the other party is full of impatience and perfunctory attitude	
	During the online communication with the other party, the other party is really online, but it takes a long time to contact me	
	I was in a bad mood and sought comfort from the other party online, but the other party replied me with some perfunctory words	
	I chatted with the other party online and had difficulty getting a warm response	
	I encountered problems and difficulties in consulting with each other online, and got no feedback	
	I send notice in network group chat room (QQ group, WeChat group, etc.), they do not reply	
	When I was upset, I seek help in the Internet chat room and they do not reply	
	When I encounter problems or difficulties, I seek comfort in the Internet group chat room, and they ignore me	
	In network group chat room where people participate in discussions, I cannot get a response to what I say as much as what they say	
	When I encounter problems and difficulties, I ask for help on my personal space on the Internet and I don’t get any help	
	I receive negative comments when I post sayings, logs, photos, etc. on my online personal space	
	Commenting on each other’s dynamics on the network personal space, and not getting feedback from each other	
	When I am in a bad mood, I seek comfort on my online personal space and get no comfort	
Conspicuous consumption	Before buying a product, it is essential to understand how your friends view different brands and categories	Marcoux, et al. [62], O’Cass and McEwen [63]
	Before buying a product, it is important to be clear about how others will perceive people who use the brand or category	
	Before purchasing a product, it is important to know what brand or category can make a good impression on others	
	I used to buy expensive brands just because I knew others would notice it	
	I love the prestige that comes with buying a brand	
Sense of control	I often feel helpless in the process of using social media	Niemeyer, et al. [64]
	In the process of using social media, there are many things that happen that are often out of my control	
	There are many things that get in the way of what I want to do in using social media	
Social media use intensity	Social media is a part of my daily activities	Ellison, et al. [65]
	When I use social media, I am proud to tell others	
	I feel disconnected from the outside world because I haven’ t logged on to social media for a while	
	I feel like I’ m integrated into the social media community	
	I would feel bad if social media was blocked	
Implicit personality	People are born with their types and are hard to change	Levy, et al. [66]
	Although people may do things differently, their nature does not change	
	Everyone is a person of a particular personality type, and it is difficult to change personality	
	As much as I hate to admit that habits are hard to change, it’s true that people can’t really change their deepest traits	
	People can change even their most fundamental traits	
	People can transform themselves into a different personality type altogether	
	Everyone can significantly change their basic personality traits	
	No matter which personality type an individual is, they can often undergo large changes	

**Table 4:** Means, standard deviations, and correlation analysis.

	Variables	Mean	SD	1	2	3	4	5	6	7	8	9
1	Gender	0.563	0.504	-								
2	Age	32.248	9.536	−0.032	-							
3	Education level	16.825	1.767	−0.086	0.034	-						
4	Monthly living expenses	3.864	1.482	−0.025	0.217 *	0.036	-					
5	Cyber-ostracism	3.333	1.131	0.042	0.028	−0.012	−0.324 **	-				
6	Conspicuous consumption	3.779	0.818	0.024	−0.012	0.016	0.216 *	0.462 **	-			
7	Sense of control	2.581	1.060	0.012	0.031	0.029	−0.115	−0.632 **	−0.481 **	-		
8	Social media use intensity	3.692	0.996	0.036	0.148	0.174	0.129 *	0.064	0.241 **	−0.018	-	
9	Implicit personality	0.287	1.486	0.026	0.035	−0.043	0.113	0.125 *	−0.043	−0.075	0.042	-

Note. *N* = 407. * *p* < 0.05, ** *p* < 0.01.

**Table 5:** Model fit statistics for measurement models.

Model	χ2 (df)	CFI	TLI	RMSEA	SRMR	Δχ2 (df)
Theoretical five-factor model(CO, CC, SC, SM, IP)	393.446 (254)	0.975	0.972	0.037	0.042	
Four-factor model(CO and CC, SC, SM, IP)	782.382 (258)	0.886	0.872	0.052	0.073	373.226(4) ***
Three-factor model(CO and CC, SC and SM, IP)	1472.604 (262)	0.792	0.769	0.094	0.124	1129.886(5) ***
Two-factor model(CO and CC and SC and SM, IP)	1825.863 (264)	0.683	0.677	0.112	0.145	1358.323(7) ***
One-factor model(CO and CC and SC and SM and IP)	2120.334 (272)	0.595	0.584	0.185	0.168	1522.306(8) ***

Note. CO: Cyber-Ostracism, CC: Conspicuous consumption, SC: Sense of control, SM: Social media use intensity, IP: Implicit personality; *** *p* < 0.001.

**Table 6:** Results of the hierarchical regression analysis.

Variables	Model 1			Model 2			Model 3		
	Conspicuous Consumption			Sense of Control			Conspicuous Consumption		
	β	SE	*t*	β	SE	*t*	β	SE	*t*
Gender	0.005	0.089	0.057	0.011	0.076	0.140	0.008	0.086	0.097
Age	0.051	0.064	0.795	0.079	0.055	1.436	0.075	0.062	1.208
Education level	−0.037	0.054	−0.694	0.019	0.046	0.402	−0.032	0.052	−0.610
Monthly living expenses	0.155 *	0.063	2.455	−0.233 ***	0.054	−4.282	0.084	0.062	1.351
Cyber-ostracism	0.406 ***	0.050	8.198	−0.569 ***	0.043	−13.387	0.232 ***	0.058	4.040
Sense of control							−0.304 ***	0.056	−5.423
*R^2^*	0.226			0.428			0.279		
Δ*R^2^*	0.084 ***			0.062 ***			0.167 ***		
*F*	23.445 ***			59.993 ***			25.822 ***		

Note. *N* = 407; * *p* < 0.05, *** *p* < 0.001.

**Table 7:** Tests for moderating effects.

Variables	Sense of Control						Conspicuous Consumption					
	Model 1		Model 2		Model 3		Model 4		Model 5		Model 6	
	β	*t*	β	*t*	β	*t*	β	*t*	β	*t*	β	*t*
Gender	−0.039	−0.847	0.005	0.140	0.005	0.139	0.006	0.160	0.022	0.013	0.005	0.104
Age	−0.041	−0.886	0.056	1.435	0.055	1.382	0.054	1.373	0.034	0.047	0.103	0.015
Education level	0.015	0.313	0.016	0.402	0.016	0.398	−0.002	−0.062	0.023	0.322	0.214	0.026
Monthly living expenses	−0.409 ***	−8.677	−0.183 ***	−4.282	−0.183 ***	−4.282	−0.177 ***	−4.171	0.128 ***	0.132	0.226 ***	0.106
Cyber-ostracism			−0.233 **	−6.386	−0.337 ***	−5.342	0.247 ***	3.221				
Social media use intensity					0.020	0.537						
Sense of control									−0.119 ***	0.116		
Implicit personality											0.113	0.025
Cyber-ostracism*Social media use intensity					−0.135 ***	−0.216						
Sense of control*Implicit personality											−0.154 ***	−3.588
Cyber-ostracism*Implicit personality			0.128						0.173			
*R^2^*	0.172		0.375		0.428		0.442		0.239		0.144	
Δ*R^2^*	0.084 ***		0.062 ***		0.167 ***		0.058 ***		0.031 ***		0.072 ***	
*F*	20.919 ***		59.993 ***		49.953 ***		45.121 ***		33.556 ***		20.919 ***	

Note. *N* = 407; *** *p* < 0.001.

**Table 8:** Moderated mediation results for a conditional indirect effect.

Task	Boot Indirect	Boot SE	95% of Confidence Intervals	
Complexity	Effect (β)		Boot LLCI	Boot ULCI
Low level (−1 SD)	−0.6782	0.0551	−0.3033	−0.1384
Mean	−0.5643	0.0423	−0.4272	−0.2092
High level (+1 SD)	−0.4503	0.0572	−0.1068	−0.0243

**Table 9:** Moderated mediation results for a conditional indirect effect.

Task	Boot Indirect	Boot SE	95% of Confidence Intervals	
Complexity	Effect(β)		Boot LLCI	Boot ULCI
Entity theorist (−1 SD)	−0.3427	0.0674	−0.2217	−0.0391
Mean	−0.3183	0.0582	−0.4552	−0.2585
Incremental theorist (+1 SD)	−0.1712	0.0741	−0.1856	−0.0218

## Data Availability

The data of this study are available from the corresponding author upon reasonable request.
